# Host adaptation in *Salmonella enterica* serovar Typhimurium: population structure, pathovariants, and genomic mechanisms

**DOI:** 10.1128/aem.02201-25

**Published:** 2026-02-10

**Authors:** Hairuo Gong, Qiuhui Wu, Mengnan Xu, Wenzhe Meng, Yunfei Wang, Changyan Ju, Yezhi Fu

**Affiliations:** 1 School of Agriculture and Biotechnology, Sun Yat-sen University, Shenzhen, China; 2 Nanshan District Center for Disease Control and Prevention 681100 , Shenzhen, China; 3 Guangdong Engineering Technology Research Center of Nutrition Transformation, Sun Yat-sen University, Shenzhen, China; Universidad de los Andes, Bogotá, Colombia

**Keywords:** *Salmonella*Typhimurium, whole-genome sequencing, host adaptation, pathovariant, genome degradation

## Abstract

*Salmonella enterica* serovar Typhimurium is a major zoonotic pathogen of global concern to human and animal health. With its broad host range, this serovar can colonize humans as well as domesticated and wild animals. Although historically considered a model host-generalist pathogen, whole-genome sequencing (WGS) has uncovered substantial genetic diversity and the emergence of multiple host-adapted pathovariants within this serovar. In this minireview, we delineate the population structure of *S*. Typhimurium across diverse host species and identify the lineages/pathovariants specifically adapted to avian hosts (e.g., passerines, pigeons, ducks, geese, larids, and water birds) and those adapted to non-avian hosts (e.g., humans). We further discuss the genetic mechanisms underlying host adaptation of *S*. Typhimurium pathovariants, including genome degradation through point mutations and insertions/deletions, as well as the acquisition of prophages or antimicrobial resistance genes via horizontal gene transfer. The ongoing emergence of host-adapted pathovariants in zoonotic pathogens such as *S*. Typhimurium underscores the importance of high-resolution, WGS-based subtyping approaches for precise pathogen identification and source attribution. Moreover, elucidating the genetic mechanisms driving host adaptation of zoonotic pathogens at the strain level is essential for informing targeted strategies for surveillance, prevention, and control.

## INTRODUCTION

*Salmonella enterica* causes millions of infections worldwide each year, leading to significant morbidity and mortality in both humans and animals (1, 2). Understanding the genetic diversity and host adaptation of this pathogen is crucial for effective disease control and prevention. The species *Salmonella enterica* encompasses over 2,500 serovars, differentiated by unique combinations of O antigen (lipopolysaccharide) and H antigen (flagella) (3). Based on host range, *Salmonella enterica* serovars are broadly classified into three categories: host-generalist serovars, host-adapted serovars, and host-restricted serovars (4). For example, *S. enterica* serovars Typhimurium and Enteritidis are generalists that can infect a wide array of hosts, including humans, livestock, poultry, and wildlife. In contrast, serovars such as Choleraesuis, Dublin, and Abortusovis are adapted to pigs, cattle, and sheep, respectively, while serovars Typhi and Paratyphi A are restricted to humans and higher primates, and serovar Gallinarum is restricted to poultry (5–7). Serovars with broad or narrow host range usually exhibit different pathogenicity and transmission ability. Specifically, host-generalist serovars typically remain within the host’s intestine, causing non-invasive diseases such as gastroenteritis, which are often acute and self-limiting. Conversely, host-adapted or host-restricted serovars can breach the host epithelial barrier and disseminate to systemic sites, leading to more severe invasive diseases (7). For example, *S*. Gallinarum is associated with fowl typhoid in poultry, *S*. Abortusovis targets the placenta and leads to abortion in sheep, and *S*. Dublin and *S*. Choleraesuis are associated with bacteremia in cattle and pigs, respectively (7).

As a host-generalist serovar, *S*. Typhimurium has long been regarded as a model organism for studying broad-host-range *Salmonella enterica* (8). Historically, research has focused primarily on a limited number of reference strains, such as SL1344, LT2, and ATCC14028 (8). However, the limited variation observed among these reference strains does not accurately reflect the genetic diversity within this serovar. Accumulating evidence indicates that this host-generalist serovar has undergone adaptive evolution within specific host species, particularly in wild birds (9, 10). Certain avian host-adapted *S*. Typhimurium pathovariants, identified through phage typing, include definite phage type (DT) 2 and DT99 in pigeons (11, 12), DT8 in ducks and geese (13), and DT40/DT56(v) and DT160 in passerines (14). In recent years, using whole genome sequencing (WGS)-based approaches, researchers identified *S*. Typhimurium pathovariants adapted to humans, larids (e.g., gulls and terns), and water birds (e.g., cormorants, pelicans, and herons), respectively (15–17). The emergence of these host-adapted pathovariants within a serovar known for its broad host range suggests that categorizing bacterial pathogens as generalists at the species or serovar level is an oversimplification. In modern outbreak investigation and infection control, the within-species or within-serovar genetic diversity and host adaptation of bacterial pathogens should be considered, as host-adapted pathovariants may present distinct pathogenicity and transmission routes.

In this review, we examine the host-associated population structure and major host-adapted pathovariants of *S*. Typhimurium and discuss the genetic mechanisms that drive its microevolution toward an adaptive lifestyle. Using this otherwise generalist serovar as a case study, we illustrate how bacterial pathogens traditionally regarded as generalists can diversify into closely related lineages or pathovariants exhibiting host-specific adaptations at the strain level. We further emphasize that the high resolution afforded by WGS enables the precise discrimination of such host-adapted pathovariants, offering valuable new insights into the genetic basis of bacterial host adaptation.

## POPULATION STRUCTURE OF *S.* TYPHIMURIUM

Host-adapted pathovariants of *S*. Typhimurium have long been recognized via traditional subtyping techniques (e.g., phage typing and pulsed-field gel electrophoresis) and well documented in the literature and epidemiological reports (13). However, comprehensive phylogenetic and genomic analyses remained limited until WGS became a routine tool for *S*. Typhimurium surveillance. The large number of genomes now available in public repositories enables population genomics studies on a global scale. Two pivotal WGS-based studies have clarified the population structure of *S*. Typhimurium across multiple host species, providing insights into its within-serovar diversity, host adaptation, and evolutionary dynamics (9, 18). Bawn et al. (18) analyzed public health WGS data of *S*. Typhimurium from the United Kingdom and identified two major clades (α and β) on the phylogenetic tree. Clade α comprises subclades such as ST34, DT104, DT193, and U288, which include strains from well-characterized epidemics in domesticated animals. In contrast, clade β encompasses subclades such as DT40/DT56(v), DT2, DT8, and DT99, which are primarily associated with wild avian species. The two clades also differ markedly in their antimicrobial resistance (AMR) profiles: livestock-associated clade α isolates often exhibit antibiotic resistance, whereas wild avian-associated clade β isolates rarely carry AMR determinants (18). Additionally, subclades in clade β display genomic signatures of host adaptation, such as the accumulation of hypothetically disrupted coding sequences in virulence genes (18). Collectively, these findings suggest that diverse avian host-adapted pathovariants originated from a common *S*. Typhimurium ancestor while still retaining the capacity to circulate among multiple livestock species.

**Fig 1:**
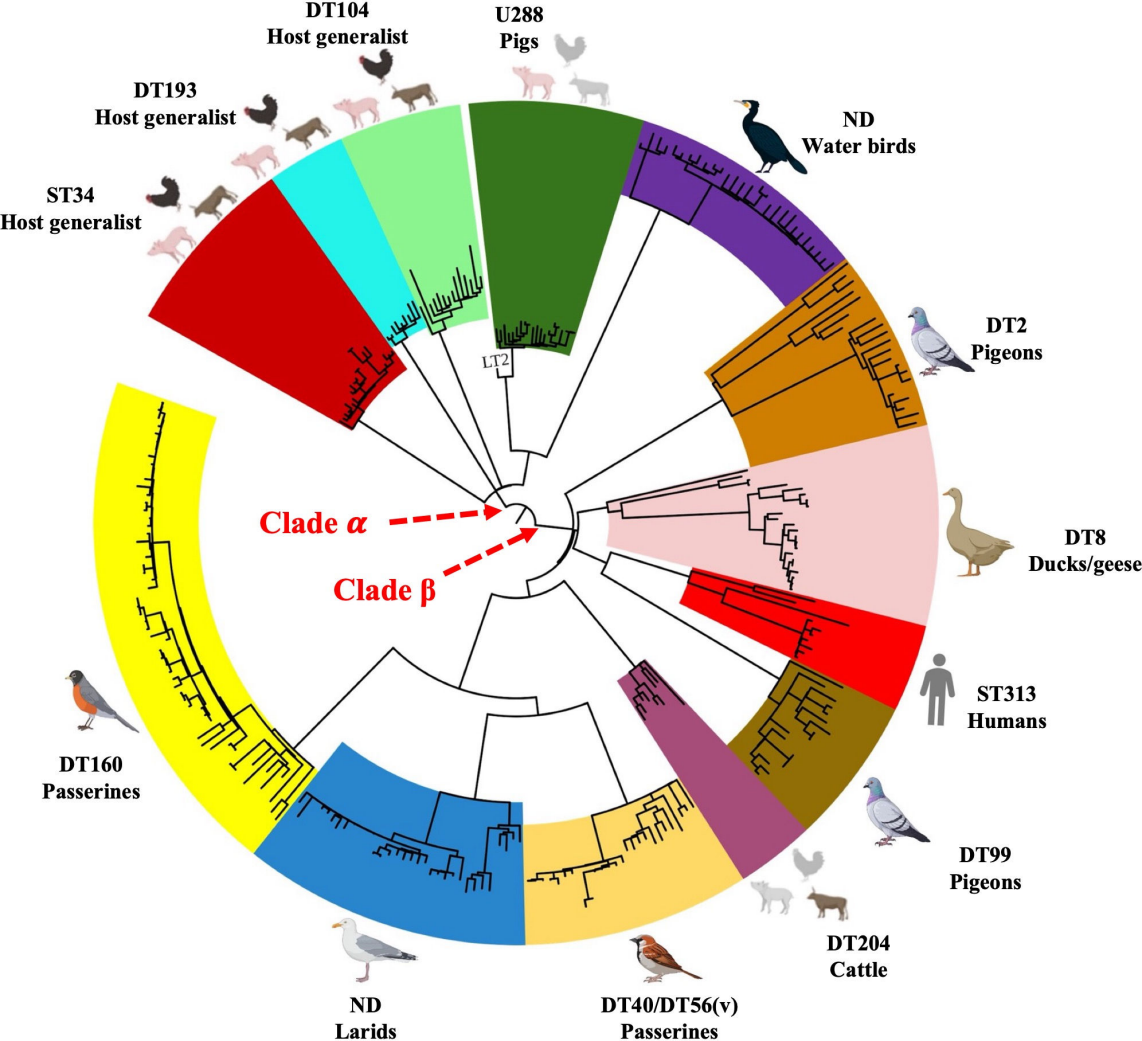
Population structure of *Salmonella enterica* serovar Typhimurium isolates derived from diverse host species based on a maximum likelihood phylogenetic tree constructed from core genome single-nucleotide polymorphisms (SNPs). The phylogeny is divided into two primary clades: clade α and clade β. Clade α comprises lineages/pathovariants exhibiting either broad or narrow host ranges, whereas clade β consists exclusively of host-adapted lineages/pathovariants. The predominant phage type/sequence type and principal host species for each lineage are indicated in the figure legend. “ND” denotes undetermined phage type. Gray shading for host species associated with the U288 and DT204 lineages indicates that these pathovariants are primarily linked to livestock, with U288 predominantly found in pigs and DT204 in cattle. Figure adapted from reference (9).

While this framework significantly advanced the understanding of *S*. Typhimurium population structure, further delineation of avian-associated diversity and expansion of geographic breadth remained needed to fully capture the global evolutionary landscape of the serovar. To address these knowledge gaps, Fu et al. (9) sequenced hundreds of *S*. Typhimurium isolates from diverse avian hosts—including passerines (order *Passeriformes*), larids (order *Charadriiformes*), ducks and geese (order *Anseriformes*), pigeons (order *Columbiformes*), and water birds (clade *Aequornithes*)—collected between 1978 and 2019 (9). By combining this data set with avian *S*. Typhimurium genomes deposited by other groups in public repositories such as EnteroBase and NCBI, the authors reconstructed the global population structure of avian host-associated *S*. Typhimurium. Phylogenetic analyses using core-genome single-nucleotide polymorphism (cgSNP) and whole-genome MLST (wgMLST) identified seven avian host-adapted *S*. Typhimurium lineages/pathovariants (9). These lineages are globally distributed and have been implicated in outbreaks of salmonellosis in both wild birds and humans (19–24). Consistent with Bawn and colleagues’ work, two major clades were identified on our phylogenetic tree when avian and non-avian *S*. Typhimurium genomes were analyzed together (Fig. 1). Notably, a previously unrecognized water bird-adapted pathovariant that lacked AMR genes was detected within clade α. This finding challenges the traditional view that clade α is exclusively associated with domesticated animals and uniformly host-generalist (9). Instead, it indicates that clade α also harbors lineages exhibiting clear host-specific adaptation, highlighting a more complex and dynamic pattern of host adaptation in *S*. Typhimurium than previously appreciated. Meanwhile, clade β consisted predominantly of avian host–adapted lineages, including those previously defined by phage typing, such as DT2 and DT99 (pigeon-adapted), DT8 (duck/goose-adapted), and DT40/DT56(v)/DT160 (passerine-adapted). In addition, it included a newly identified larid-associated lineage that has not yet been assigned a phage type (9). Beyond these dominant avian host-adapted lineages, clade β also contains two non-avian host-adapted lineages: ST313 adapted to humans and DT204 adapted to cattle (9). Comparative genomic analyses further revealed that host-adapted lineages in both clades α and β share a common “signature of adaptation” in their virulence-associated genomes, characterized by degradation of both type III secretion systems (T3SS) and adhesins. For example, all host-adapted lineages harbor pseudogenized long polar fimbriae (LPF) genes (*lpfC* and *lpfD*) due to deletion mutations (9). In addition, non-synonymous SNPs and deletions in T3SS effector gene (e.g., *sseL*, *sifB*, *sopD2*, *sseJ*, *steC*, *slrP*, and *sseK2*) are prevalent in host-adapted lineages but occur much less frequently in host-generalist lineages (9). Bayesian inference indicated that the most recent common ancestor of host-adapted pathovariants of *S*. Typhimurium likely emerged approximately 200 years ago, with an estimated evolutionary rate of ~10⁻⁷ substitutions/site/year (9). This is substantially higher than the long-term evolutionary rates (10⁻¹⁰~10⁻⁹ substitutions/site/year) reported for *Escherichia coli* or *Salmonella* (25). These findings collectively point to rapid diversification of *S*. Typhimurium pathovariants, potentially driven by anthropogenic influences such as intensified antibiotic use and increased global ecological connectivity.

## HOST-ADAPTED *S.* TYPHIMURIUM PATHOVARIANTS

### *S*. Typhimurium pathovariants adapted to avian hosts

Most host-adapted pathovariants of *S*. Typhimurium have been detected in avian hosts (Table 1). Prior to the advent of WGS, potential avian host-adapted pathovariants were identified primarily through phage typing, including DT40/DT56(v) and DT160 in passerines, DT2 and DT99 in pigeons, and DT8 in ducks and geese (13, 26). These phage types provided early evidence of host-specific associations in *S*. Typhimurium, although the underlying genetic determinants of adaptation remained unresolved. More recently, using WGS-based cgSNP or wgMLST approaches, researchers have identified *S*. Typhimurium pathovariants associated with larids (gulls and terns) and water birds that have not been reported in previous epidemiological surveys and have not been assigned to any phage type (9, 17). Epidemiological and genomic evidence collectively indicate that birds serve as reservoirs for emerging pathovariants of *S*. Typhimurium (10). These avian host-adapted pathovariants pose a potential risk for cross-species transmission and zoonotic spillover, underscoring the need for integrated surveillance and genomic characterization to track their evolution and dissemination.

**TABLE 1:** Host-adapted pathovariants of *Salmonella enterica* serovar Typhimurium

Phage type	Sequence type	Major host	Reference(s)
DT2	ST128	Pigeons	(12, 27)
DT99	ST19	Pigeons	(12, 27)
DT160	ST19	Passerines	(17, 24, 28)
DT40/DT56(v)	ST19 (main), ST568 (minor)	Passerines	(14, 19, 29)
ND^*a*^	ST19	Larids	(17)
DT8	ST19	Ducks/geese	(30, 31)
ND	ST99 (main), ST3719 (minor)	Water birds (e.g., cormorants, pelicans, and herons)	(17)
ND	ST313	Humans	(32, 33)
DT204	ST19	Cattle	(34)
U288	ST19	Pigs	(35)

^*a*^ ND, not determined.

DT40, DT56(v), and DT160 are well-characterized *S*. Typhimurium pathovariants associated with passerine birds, including finches, sparrows, siskins, and redpolls. These pathovariants have been responsible for salmonellosis outbreaks in both wild birds and humans over recent decades. DT40 and DT56(v) are most frequently reported in Europe, particularly in the United Kingdom (14, 19, 29), while DT160 has been linked to outbreaks in the United States (17, 24, 28), New Zealand, and Australia (20, 21). Comparative genomic and Bayesian phylogenetic analyses indicate that European DT40/DT56(v) and U.S. DT160 represent two distinct lineages that share a common ancestor, while Oceanian DT160 isolates form a sublineage within the U.S. DT160 lineage (23). Both lineages exhibit similar genomic signatures, including loss of the virulence plasmid pSLT, accumulation of pseudogenes in fimbrial and T3SS effector genes, and absence of AMR genes (36). Nonetheless, microevolutionary differences between the two lineages are evident. For example, the virulence plasmid pSLT is absent from all examined DT40/DT56(v) isolates but has been lost only in a subset of DT160 isolates. Furthermore, pseudogenization of the type one fimbrial gene *fimC* is unique to DT160 passerine isolates from the United States and Oceania, whereas single-nucleotide deletions in the T3SS effector genes *gogB*, *sseJ*, and *sseK2* occur exclusively in European DT40/DT56(v) isolates (23). Phylogenetic evidence further suggests that passerine-adapted *S*. Typhimurium pathovariants in the United States and Europe share a recent common ancestor, with intercontinental dissemination potentially facilitated by migratory gulls and terns (37).

DT2 and DT99 are phage types predominantly isolated from pigeons in Europe, North America, and Asia. Global phylogenetic analyses indicate that DT2 and DT99 represent two distinct *S*. Typhimurium lineages, corresponding to sequence types (STs) 128 and ST19, respectively, with the United States identified as a potential geographic origin for both pathovariants (27). Both ST128-DT2 and ST19-DT99 exhibit strong host adaptation to pigeons and are closely associated with systemic disease (pigeon paratyphoid) in this host, but not in other animals (13). This host specificity is linked to the pseudogenization of selected virulence and metabolic genes, as well as transcriptional downregulation of genes involved in motility, flagellar biosynthesis, and chemotaxis (11, 12). For example, DT2 94-213, a representative DT2 strain that causes severe systemic disease in pigeons, contains 22 pseudogenes that are intact in other generalist *S*. Typhimurium strains such as *S*. Typhimurium LT2, SL1344, and DT104 (12). In particular, the inactivation of *gtgE* and *pcgL* has been implicated in host restriction and enhanced hyperdissemination of DT2 strains (12). Although both DT2 and DT99 show evidence of pigeon-specific adaptation and harbor markedly fewer AMR genes compared to generalist *S*. Typhimurium lineages, experimental infections in mammalian and avian models indicate that DT2 exhibits a higher degree of pigeon tropism than DT99 (27). In contrast, DT99 carries a higher burden of AMR genes than DT2, likely due to a greater prevalence of plasmid-associated resistance determinants (27).

Other pathovariant groups associated with specific avian hosts include DT8, which is adapted to ducks and geese and has been implicated in foodborne outbreaks linked to the consumption of duck eggs in the United Kingdom and Ireland (30, 31). Additionally, distinct *S*. Typhimurium lineages adapted to larids and water birds have been identified; however, these two lineages have so far only been reported in North America and have not been assigned phage types (17, 24, 38). Notably, phylogenetic analyses suggest that passerine-adapted *S*. Typhimurium pathovariants, specifically DT40/DT56(v) in Europe and DT160 in North America, share a common ancestor with the larid-adapted lineage (37). The two passerine-associated lineages circulating in Europe and North America are therefore closely linked evolutionarily, and their intercontinental dissemination is hypothesized to have been facilitated by larids such as gulls and terns (37). Together, these observations highlight the interconnected evolutionary trajectories of larid- and passerine-associated pathovariants and provide a more comprehensive perspective on the host-associated population structure of *S*. Typhimurium within clade β.

### *S*. Typhimurium pathovariants adapted to non-avian hosts

ST313 is a notable variant of *S*. Typhimurium that has adapted to humans, particularly in sub-Saharan Africa (32, 33). This pathovariant is associated with invasive non-typhoidal *Salmonella* (iNTS) infections, which are often severe and life-threatening. Unlike other host-generalist *S*. Typhimurium strains that cause gastroenteritis, ST313 strains are more likely to cause bloodstream infections (32). Host adaptation of ST313 to humans is linked to genome degradation, where certain genes involved in biofilm formation, stress resistance, and gut-specific colonization and metabolism are lost (16). As a result, ST313 strains show impaired biofilm formation, reduced survival in the gastrointestinal tract, and an increased ability to spread beyond the epithelial barrier (16, 39). The genome degradation events that generate functionally relevant pseudogenes suggest that ST313 is transitioning from an intestinal to a more systemic lifestyle (16). Recent WGS analysis reveals that antibiotic usage to treat iNTS infections in sub-Saharan Africa has significantly influenced the microevolution of ST313, resulting in the emergence of distinct ST313 sublineages, each characterized by unique AMR profiles (16, 40, 41).

Host-adapted *S*. Typhimurium pathovariants have also been identified in livestock. Phage types U288 and DT204 are predominantly isolated from pigs and cattle, respectively, and only rarely from humans since their emergence, suggesting a degree of host adaptation to these livestock hosts (34, 35). Notably, similar to *S*. Choleraesuis, a serotype highly adapted to pigs, the *pncA* gene is disrupted in *S*. Typhimurium U288, representing a key molecular signature of this phage type (35). Furthermore, several genes disrupted in U288 but intact in ST34 are associated with colonization of the porcine intestine, consistent with the observation that U288 infections disseminate more readily to swine lymph nodes rather than remaining confined to the gut (35).

## GENETIC MECHANISMS OF HOST ADAPTATION OF *S.* TYPHIMURIUM

Historically, knowledge of *S*. Typhimurium pathovariants has been derived primarily from traditional subtyping methods and epidemiological investigations, leaving the genetic basis of their emergence largely unresolved (8). The advent of WGS now enables high-resolution comparative genomic analyses of *S*. Typhimurium populations across multiple hosts, providing critical insights into the genetic mechanisms underlying host adaptation—such as genome degradation via point mutations and the acquisition of AMR genes through horizontal gene transfer (HGT).

### Genome degradation via point mutations or indels

Mutations leading to gene inactivation or pseudogenization, including point mutations and indels, are central to the evolutionary host adaptation of *Salmonella* (42). For instance, compared with host-generalist serovar *S*. Enteritidis, host-restricted serovar *S*. Gallinarum has accumulated numerous pseudogenes associated with fimbrial structures throughout its evolutionary history. This pseudogenization has led to the loss of functional fimbriae, thereby limiting its ability to survive in the external environment and promoting a transition from environmental transmission to persistent colonization within poultry hosts (43). Moreover, T3SS effector genes that typically remain intact in host-generalist *Salmonella* serovars are often pseudogenized in host-adapted serovars. This suggests that these virulence factors may no longer be essential for infection in specific hosts and that their inactivation may actually facilitate adaptive colonization and immune evasion (44). Recent studies have further shown that pseudogenization of fimbrial and T3SS effector genes occurs not only among different *Salmonella* serovars but can also drive host adaptation within the same serovar (45, 46). In particular, host-adapted pathovariants of *S*. Typhimurium have undergone parallel patterns of genome degradation, likely facilitating a shift from a broad to a restricted host range (9, 18). For example, a 10-bp deletion leading to pseudogenization of the long polar fimbriae (LPF) gene *lpfD* has been identified in nearly all host-adapted *S*. Typhimurium pathovariants, with the notable exception of the water bird-adapted lineage. In contrast, the water bird-adapted pathovariants harbor a single-base deletion causing pseudogenization of *lpfC* (9). These findings indicate that different components of the LPF system may independently undergo functional degradation during host adaptation, rather than host adaptation being driven by a single conserved mutation event. Given that LPF mediates adhesion to intestinal M cells and promotes translocation across the gut epithelium (47, 48), such pseudogenization may reduce intestinal colonization while facilitating immune evasion and systemic dissemination (49, 50). Collectively, these observations suggest that pseudogenization of distinct LPF genes represents a broader and more flexible evolutionary strategy underlying host adaptation in *S*. Typhimurium, a perspective that has not been explicitly emphasized in previous reviews. In addition to fimbrial genes, T3SS effector genes such as *sseL*, *sifB*, *sopD2*, *sseJ*, *steC*, *slrP*, and *sseK2* also constitute hotspots for pseudogenization in *S*. Typhimurium pathovariants, although the specific effector genes affected differ among lineages (9). T3SS effectors are central to bacterial invasion of non-phagocytic cells, modulation of host inflammatory responses, and survival within phagocytes (44); therefore, their pseudogenization in host-adapted *S*. Typhimurium pathovariants may reflect relaxed selection for intestinal invasion and a shift toward persistent systemic colonization in specific hosts (27, 51). Notably, despite genome degradation through point mutations or indels in fimbrial and T3SS genes, the overall virulence gene repertoire of host-adapted pathovariants remains largely comparable to that of host-generalist *S*. Typhimurium, and no other consistent virulence gene mutations or novel virulence genes have been identified across distinct host-adapted lineages (9). Collectively, these findings suggest that *lpf* pseudogenization, along with lineage-specific loss of T3SS effector genes, may represent a key genetic mechanism driving the adaptive evolution and pathogenesis of host-adapted *S*. Typhimurium pathovariants.

### Horizontal gene transfer

In addition to pseudogenization of virulence-associated genes, the acquisition of prophages and/or AMR genes through horizontal gene transfer (HGT) represents another important mechanism contributing to the emergence of *S*. Typhimurium pathovariants. However, unlike genome degradation caused by point mutations or indels, which is more directly linked to host specificity, this mechanism primarily reflects the microevolutionary processes shaping *S*. Typhimurium pathovariant diversification. For instance, *S*. Typhimurium ST313 has evolved into three lineages due to microevolution via HGT. ST313 lineages 1 and 2 harbor five full-length prophages: Gifsy-2, ST64B, and Gifsy-1—commonly found in many *S*. Typhimurium genomes—along with two novel prophages, BTP1 and BTP5 (52). Notably, BTP1 encodes the anti-virulence gene *bstA*, which is also widely present in isolates of the bovine-adapted serovar *S*. Dublin (53, 54). In contrast, ST313 lineage 3 lacks BTP1 and BTP5 but instead harbors a P22-like prophage and a Fels-2-like prophage. Comparative genomic analyses show that most virulence genes specific to lineage 3, relative to lineages 1 and 2, are prophage- or plasmid-encoded. Moreover, unlike lineages 1 and 2, which carry a multidrug resistance cassette on the pSLT-BT plasmid, lineage 3 lacks resistant plasmids and remains pan-susceptible to all antimicrobials tested, both genotypically and phenotypically (16). The distinct prophage and plasmid repertoire of lineage 3, combined with the degradation of key virulence genes absent in other ST313 lineages, may enhance its ability to cause extraintestinal infections while limiting its antimicrobial resistance (16).

## CONCLUSION AND OUTLOOK

Each host species represents a distinct ecological niche for bacterial pathogens. During colonization and infection, pathogens must overcome multiple challenges, including host immune defenses, antimicrobial treatments, and competition from the resident microbiota (55). These selective pressures drive bacterial evolution within the host environment, promoting genomic changes that facilitate immune evasion and AMR, ultimately leading to the emergence of novel pathovariants within the same species (56–58). An in-depth investigation of host-adaptive evolution in zoonotic pathogens and their emerging pathovariants is therefore essential for elucidating disease origins, potential transmission pathways, and the risks associated with pathogenicity and AMR. Such insights provide a robust scientific basis for pathogen source tracing and the development of targeted prevention and control strategies within a “One Health” framework, thereby contributing to the protection of both human and animal health. In this review, we summarize new insights into the host-adaptive evolution of *S*. Typhimurium by emphasizing its host-associated population structure and the emergence of lineages/pathovariants with distinct host preferences. From a genomic perspective, we further show that functional degradation of virulence-associated genes, driven by point mutations or indels, represents a major evolutionary mechanism underpinning the diversification of *S*. Typhimurium pathovariants.

The continuous emergence of host-adapted pathovariants in zoonotic bacterial pathogens such as *S*. Typhimurium underscores the need for high-resolution subtyping approaches, including WGS-based cgSNP analysis and MLST schemes, to enable precise strain-level discrimination. It further highlights the importance of strengthening One Health-oriented genomic surveillance frameworks that integrate genomic data of zoonotic bacterial pathogens not only from humans, domesticated animals, and food sources but also from wildlife, particularly wild birds. In addition, research on *S*. Typhimurium pathovariants has thus far predominantly focused on epidemiological investigations and genomic analyses, whereas studies addressing their adaptive evolutionary mechanisms remain limited. Notably, most candidate host-adaptive mutations identified to date are based on population genomic inference and still await experimental validation. Moving forward, future research should systematically investigate the molecular mechanisms underlying host adaptation and persistence, with particular emphasis on the key genetic changes that drive host specificity in *S*. Typhimurium. For example, the potential role of *lpfC and lpfD* gene pseudogenization in driving host range contraction and tissue tropism shifts in *S*. Typhimurium remains to be experimentally validated.
